# Spontaneous Evolution of the Fœtus

**Published:** 1832

**Authors:** S. C. Snyder

**Affiliations:** Charleston, Va.


31. *Spontaneous Evolution of the Fœtus.* By S. C. Snyder, of Charleston, Va.—When Dr. S. first saw the patient, the labor had commenced, and the liquor amnii escaped. The child was found with the left shoulder resting upon the left acetabulum and the body stretched across the pelvis of the mother. After some delay in consequence of waiting for a consulting physician, the os uteri being well dilated and the funis still pulsating, an attempt was made to turn the child, but the hand having been introduced into the vagina, so instantaneous and violent were the contractions of the uterus on the least attempt to move the child, that the consultation, apprehensive of a rupture of the uterus, determined to wait for a spontaneous evolution. That this would take place they inferred from the capacity of the pelvis, the size of the child, the activity of the womb, and the health of the mother.

Soon after the attempt to turn, the funis ceased to pulsate, and the shoulder and arm, not long after were protruded entirely beyond the vagina, and as the left side and pelvis of the child descended the long diameter of the mother's pelvis, they gradually turned so that when the child became visible, its back as well as side presented, (the arm and shoulder, however, still remaining without). The index fingers were now passed around the groin, and the pelvis and lower extremities easily extracted; the head followed; soon after, the placenta was easily removed, but sufficient hæmorrhage followed to call for the tampon, cold wet clothes, and a large dose of sach. saturni.

The labor continued about ten hours after Dr. S. first saw the patient, with a constant bearing down of the womb, such as occurs after the operation of ergot.

Can it ever be proper for a practitioner, under the circumstances enumerated above, to attempt turning without venesection, and especially without the administration of a large anodyne? Could it be justifiable, under circumstances of such excessive action of the uterus, to wait ten hours for the spontanous evolution of the fœtus with the knowledge of its certain death?

